# CORRELATES OF SUBTLE COGNITIVE DYSFUNCTION IN A HIGHLY EDUCATED COHORT OF MID LIFE AND OLDER AFRICAN AMERICANS

**DOI:** 10.1093/geroni/igad104.3092

**Published:** 2023-12-21

**Authors:** Travonia Brown-Hughes, Alyssa Gamaldo, Corinne Pettigrew, Roland J Thorpe

**Affiliations:** Hampton University, Hampton, Virginia, United States; Clemson University, Clemson, South Carolina, United States; Johns Hopkins University, Baltimore, Maryland, United States; Johns Hopkins University, Baltimore, Maryland, United States

## Abstract

Remote cognitive screening modalities can be an initial step in assessing cognitive health. This cross-sectional study examined how risk and protective factors relate to cognitive performance in non-demented African Americans aged 50 and older. Participants were part of the African American-United Memory and Aging Project (AA-UMAP), a geographically diverse cohort of college-educated adults recruited and evaluated via telephone and online. Demographic, health variables and activity engagement were self-reported through an online survey. Cognition (global cognition, memory, and psychomotor/attention) was measured remotely using the Telephone Interview for Cognitive Status (TICS-40; N = 143) and the CogState Brief Battery (CBB; N = 51). Health variables included a mental health composite (e.g., depression and/or anxiety) and a metabolic conditions composite (e.g., high cholesterol, hypertension, diabetes, and/or obesity). Activity engagement was assessed with self-reported physical, mental, and social lifestyle activities. Linear regression models indicated that older age was associated with slower psychomotor/attention performance (p < 0.01) and marginally lower learning/working memory accuracy (p = 0.08) even after adjusting for covariates (e.g., sex and education). Cognitive performance was unrelated to mental health, metabolic conditions, and lifestyle activity engagement. Neither health-related risk factors nor level of engagement activities were significantly associated with cognitive performance in this remotely screened sample of highly educated middle-aged and older African American adults. More studies of midlife and older African Americans are needed to obtain a more in-depth understanding of the degree to which biopsychosocial strengths and deficits in midlife may affect or alter later-life cognitive trajectories.

